# Impact of Different Types of Diet on Gut Microbiota Profiles and Cancer Prevention and Treatment

**DOI:** 10.3390/medicina55040084

**Published:** 2019-03-29

**Authors:** Rainer J. Klement, Valerio Pazienza

**Affiliations:** 1 Department of Radiotherapy and Radiation Oncology, Leopoldina Hospital Schweinfurt, Robert-Koch-Straße 10, 97422 Schweinfurt, Germany; 2 Gastroenterology Unit IRCCS “Casa Sollievo della Sofferenza”, Hospital San Giovanni Rotondo, 71013 Foggia, Italy

**Keywords:** cancer, calorie restriction, ketogenic diet, Mediterranean diet, microbiota

## Abstract

Diet is frequently considered as a food regimen focused on weight loss, while it is actually the sum of food consumed by the organism. Western diets, modern lifestyle, sedentary behaviors, smoking habits, and drug consumption have led to a significant reduction of gut microbial diversity, which is linked to many non-communicable diseases (NCDs). The latter kill 40 million people each year, equivalent to more than 70% of all deaths globally. Among NCDs, tumors play a major role, being responsible for 29% of deaths from NCDs. A link between diet, microbiota, and cancer prevention and treatment has recently been unveiled, underlining the importance of a new food culture based on limiting dietary surplus and on preferring healthier foods. Here, we review the effects of some of the most popular “cancer-specific” diets on microbiota composition and their potential impact on cancer prevention and treatment.

## 1. Introduction

Diet (from the greek δίαιτα, dìaita, “way of life”) and dietary habits have a major impact on quality of life, health, and longevity. They are typically influenced by geographical, religious, ethics, and cultural choices [1]. Beside the diets designed for weight management, scientists are studying several food regimen approaches in order to establish healthy medical and functional diets with the right balance of vitamins, minerals, and other nutrients with the intent to prevent and cure health problems. One of the main “organs” finely affected by dietary intake is the gut microbiota. The latter is composed of roughly 3.8 × 10^13^ microorganisms of about 1.8 kg in weight [2] and includes at least 1000 different species of known bacteria with more than 3 million genes involved in several vital functions such as digestion of carbohydrates, suppression of harmful microbes (by competitive exclusion), vitamin synthesis, immune system activity, and drug metabolism [3,4]. The gut microbiota is now considered the “hidden” organ involved in the maintenance of host energy homeostasis and in the stimulation of host immunity. The crosstalk between the host and the microbiota that reside in the gut generates a homeostatic balance of bacteria beneficial to the host [5]. From the current knowledge, homeostasis associated with the healthy status of the host is referred to as “eubiosis” (from the greek eu = good and bios = life), while, in contrast, an unbalanced proportion of bacteria causing an imbalance in the microbial equilibrium is termed “dysbiosis” [5]. The healthy composition of microbiota mainly consists of Bacteroidetes and Firmicutes as the dominant bacterial phyla (~90% of the total bacteria), while the remaining 10% are partitioned between Proteobacteria, Verrucomicrobia, and Actinobacteria in most people [6]. As already reported, dysbiosis can be categorized into three different types [7]: (1) loss of beneficial microorganisms, (2) unwarranted growth of potentially harmful microorganisms, and (3) reduced overall microbial diversity. In most cases, these three types of dysbiosis are not mutually exclusive and can occur concurrently. Since dysbiosis has been implicated in a wide range of diseases including cancer [8] and since diet is one of the most important influencing factors of homeostasis of the gut microbiota, we summarize here scientific findings on the link between the most popular cancer-related diets, the microbiome, and cancer prevention and therapy. The scope of this review does not include the protozoan or multicellular eukaryotic inhabitants of the gut, whose response to the dietary patterns discussed below is much less known. However, these organisms have an influence on the microbiota and, therefore, host metabolism, and we refer the interested reader to the recent reviews by Bhattacharjee et al. [9] and Leung et al. [10].

## 2. Short-Chain Fatty Acids, Microbiota, and Cancer

**Figure 1:**
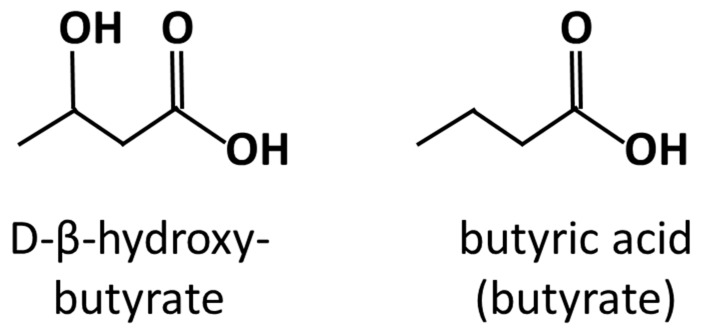
Comparison between butyrate and the physiological ketone body D-β-hydroxybutyrate.

Before we discuss particular dietary regimes, we will briefly review the effects of short-chain fatty acids (SCFAs) on cancer prevention and treatment. The microbial production of SCFAs in sufficient quantities appears to be a hallmark of health-promoting diets, at least without nutritional ketosis (see below). A major source of SCFAs in the gut is the anaerobic fermentation of polysaccharides that are indigestible for the host and, therefore, are referred to as microbiota-accessible carbohydrates or, simply, fiber. SCFAs have been shown to inhibit cancer cell growth to varying extents and through multiple mechanisms. The best investigated SCFA in this regard is butyrate, which is structurally similar to the ketone body β-hydroxybutyrate (Figure 1); indeed, both molecules also share certain physiological similarities [11]. β-hydroxybutyrate, however, has a completely different origin, stemming mainly from hepatocytes (see Section 6 below).

Sources of butyrate include [11]: (i) Direct consumption, since it is contained within certain foods (in particular, butter and cheese or human breast milk for babies); (ii) dietary fiber fermentation through butyrogenic bacteria, most of which belong to the Firmicutes phylum; (iii) fermentation of acetate and lactate obtained by cross-feeding from other microbial species; (iv) degradation of mucin (the structure-giving glycoprotein component of mucus) by certain bacteria including the Clostridia species *Rosburia intestinalis* and *Eubacterium rectaleamong*. Butyrate is the most important energy source for healthy colonocytes, accounting for roughly 70% or more of their energy requirements through β-oxidation and being preferred over ketone bodies, glucose, and glutamine [12]. In contrast, butyrate metabolism, along with oxidative phosphorylation in general, becomes impaired in pathological conditions of colitis and colon cancer, leading to a compensatory upregulation of substrate-level phosphorylation, in particular, glycolysis [13,14]. Donohoe et al. have shown that impairment of butyrate metabolism in colon cancer cells leads to its intranuclear accumulation, promoting its function as a class I and II histone deacetylase inhibitor (HDACi) which induces growth inhibition and cell death [14]. Accordingly, butyrate has been shown to inhibit proliferation and promote differentiation and apoptosis in different cancer cell lines [15,16,17]. Other chemopreventive mechanisms of butyrate include its role as a ligand for several G protein-coupled receptors (GPRs) [18]. For example, activation of the GPR109A by butyrate was shown to inhibit pro-inflammatory nuclear factor-κB signaling and tumorigenesis. The production of the endocrine gut hormones glucagon-like peptide 1 and peptide YY is also stimulated by butyrate-induced GPR activation; these hormones have important roles in satiety signaling and insulin homeostasis.

## 3. Fasting, Microbiota, and Cancer

Fasting is a dieting pattern consisting in abstinence from all solid food for a defined period. It has been practiced for millennia, especially as a religious observance, and—involuntarily—probably for much longer (millions of years) during human evolution, in this way shaping human metabolic flexibility [19]. Beyond weight loss, scientists are endorsing fasting diets, pointing to the associated health benefits [20]. Numerous scientific reports demonstrated the beneficial effects of fasting, short-term calorie restriction, or protein-restricted diets in mice models of certain types of cancer [21,22,23], accompanied by a decrease of side effects of chemotherapy in patients [21,24]. One of the main mechanism through which fasting induces metabolic improvements is certainly mediated by the gut microbiota [25]. For instance, every-other-day fasting (EODF) treatment led to a shift in the gut microbiota composition, increasing the levels of Firmicutes while decreasing most other phyla and consequently increasing the production of SCFAs as compared to control animals fed ad libitum.

Fasting promotes apoptosis in colon cancer models and induces an anti-Warburg effect that increases oxygen consumption but reduces ATP-synthesis, indicating an increase in mitochondrial uncoupling [26].

Furthermore when fasting was combined with conventional therapies (e.g., gemcitabine, temozolomide), most of the mice displayed a reduced tumor size as compared to the controls, indicating that fasting improves tumor-bearing survival and is safe, feasible, and well tolerated [23,27]. Food withdrawal decreases the abundance of potentially pathogenic Proteobacteria while increasing *Akkermansia muciniphila* levels [28]. Patients with metastatic melanoma and high levels of *A. muciniphila* are responsive to anti-PD1 therapy, indicating a role for microbiota also in immunotherapy [29].

However, fasting itself can worsen the cachexia syndrome, a medical condition which affects about half of all cancer patients. Since it has been demonstrated that the administration of probiotics and prebiotics improves the response to therapy and limit toxic side effects [30], one study tested the idea to shape the gut microbiota in order to slow down cachexia by administering *Lactobacillus reuteri* in drinking water to mice with colon cancer predisposed to cachexia. Gastrocnemius muscle masses and body weight were increased in *L. reuteri*-supplemented mice as compared to untreated mice, together with reduced neutrophil counts, a marker of systemic inflammation [31].

## 4. Mediterranean Diet, Microbiota, and Cancer

The dietetic pattern common for many populations living in the Mediterranean Basin is the so-called Mediterranean diet (MD). Besides being recognized by UNESCO as a cultural heritage of humanity, a MD improves the overall health status, reducing the risk of non-communicable diseases (NCDs) [32]. The characteristics of this dietetic pattern consist mainly of the following basics [33,34]: (a) high consumption of vegetables, fruits, cereals (mostly whole grains), nuts, and legumes; (b) low consumption of saturated fat, sweets, and meat; (c) high intake of unsaturated fat (particularly olive oil); (d) medium-high fish consumption; (e) moderate consumption of wine; (f) medium-low intake of dairy products (mainly yogurt and cheese). Features (a)–(c) are thereby considered as key characteristics for the prevention of NCDs through effects on the microbiota, as a low intake of saturated fatty acids (SFAs) and a high intake of mono- and polyunsaturated fatty acids have been associated with reduced inflammatory signaling [35], and a large intake of microbiota-accessible carbohydrates with the production of SCFAs. Consistently, higher fecal SCFA levels were found in subjects who adhered better to an MD pattern, in particular concerning the consumption of fruits, vegetables, and legumes and independent from the overall diet character (vegan, vegetarian, or omnivore) [36,37].

Besides other mechanisms such as providing a high polyphenol and micronutrient intake, the microbial characteristics of subjects adhering to an MD are therefore thought to be protective against cancer [34]. Interestingly, the abundance of the *Fusobacterium nucleatum* which is prevalent in human colorectal carcinomas and has been associated with their development, was shown to increase after only two weeks of a high-fat (52% energy), low-fiber (12 g/day) Western-style diet intervention [38].

However, the cancer-preventive effects of an MD pattern appear not to be restricted to the gut. Data from the EPIC study [39] showed that an MD pattern is associated with a 33% reduced risk of gastric cancer, and data from the MOLI-SANI study [40] found a lower level of circulating markers of inflammation such as C-reactive protein (CRP), leukocytes, platelet counts, and granulocyte/lymphocyte ratio. The latter is also associated with a poorer prognosis in cancer, acting as an independent predictor of tumor growth, progression, and metastatic processes [41]. Recently, Shivley et al. also showed that female monkeys eating an MD over 31 months experienced a shift in the microbiome of their mammary glands, most profoundly with a 10-fold increase of *Lactobacillus* abundance compared to Western diet-fed monkeys, which was accompanied by an increase in breast bile acid metabolites and a decrease in reactive oxygen species metabolites [42]. This study provides a proof-of-concept that dietary choices also affect cancer risk in organs outside the gut by modulating the local microbiota.

In conclusion, an MD pattern is associated with beneficial microbiome-related profiles inside and outside the gut and may, therefore, be an option for individuals to prevent NCDs including cancer.

## 5. Low-Carbohydrate Diet, Microbiota, and Cancer

Restricting carbohydrates consumption is an additional dietary approach associated to weight loss and improved health markers [43]. From an evolutionary perspective, a low-carbohydrate intake (<40% of energy) would have been the norm and result in cancer protection by avoiding hyperglycemia and hyperinsulinemia [44]. In overweight individuals, a low-carbohydrate, high-protein weight loss diet had no effect on the proportion of different bacterial phyla but triggered significant decreases in *Collinsella aerofaciens* and *E. rectale* relatives [45]. Instead, diets rich in complex carbohydrates increased the levels of beneficial *Bifidobacteria* such as the subspecies *Bifidobacterium longum*, *Bifidobacterium breve*, and *Bifidobacterium thetaiotaomicron* [46], while, on the other hand, reducing the growth of opportunistic species such as *Mycobacterium avium* subspecies *paratuberculosis* and Enterobacteriaceae [45].

Conversely, excessive intake of refined sugars is known to mediate noxious effects on human health, a phenomenon that is referred to as “carbotoxicity” [47], triggering the proliferation of pathogenic bacteria like *Clostridium difficile* and *Clostridium perfringens* by increasing bile output [48]. Moreover, scientific evidence suggests that sugar compounds condition the microbiota, resulting in the acquisition of a Westernized microbiome which is characterized by a substantial depletion of gut microbial diversity [49,50]. It is well recognized that tumor cells rely on glucose and glycolysis as a major source of energy, and dietary sugar intake can promote tumor formation [51]. An interesting approach to avoid carbotoxicity is the replacement of the digestible carbohydrates with resistant starch. In a mouse model of pancreatic cancer, resistant starch retarded tumor growth and simultaneously changed the microbiota profile favoring the growth of anti-inflammatory microorganisms whilst decreasing the pro-inflammatory ones [52]. Specifically, pancreatic cancer-bearing mice fed a fiber-rich food regimen displayed significantly reduced proinflammatory microorganisms such as *Bacteroides acidifaciens*, *Escherichia coli*, *Ruminococcus gnavus*, and *Clostridium cocleatum* and, on the other hand, increased levels of butyrate-producing bacteria such as Lachnospiraceae. Assuming a causal mechanism from microbiota to cancer growth, these data suggest that engineered diets with a positive influence on gut microbial communities may synergistically cooperate with standard anti-cancer therapies.

## 6. Ketogenic Diet, Microbiota, and Cancer

Ketogenic diets (KDs) are a special type of low-carbohydrate diets that reduce carbohydrate content to such an extent (usually <50 g/day) that the corresponding low insulin levels and mildly elevated cortisol levels induce the production of ketone bodies in the liver. KDs may promote metabolic health and protection against cancer and other NCDs through multiple mechanisms that include: (i) lowering insulin levels; (ii) enhancing mitochondrial substrate oxidation resulting in a sustained mild elevation of mitochondrial reactive oxygen species production and a hormetic anti-oxidative adaption; (iii) specific anti-oxidative and anti-inflammatory effects of the ketone body β-hydroxybutyrate which is a HDACi (similar to butyrate from which it differs by only a hydroxyl group, see Figure 1) [53]. However, not much is known about whether a KD can promote a healthy microbiome and gut metabolome. For cancer prevention, it could be hypothesized that a high intake of mono-unsaturated fatty acids and n-3 PUFAs as well as non-starchy vegetables would be beneficial for promoting gut health. Such a composition would be typical, e.g., for the Spanish Mediterranean KD [54]. Furthermore, given the structural and functional similarity between butyrate and β-hydroxybutyrate, it could be hypothesized that higher systemic concentrations of the latter could decrease the importance of microbial butyrate production.

In most preclinical tumor models, KDs have been shown to inhibit cancer cell glycolysis and proliferation, and some findings have also indicated the translation of such anti-tumor effects to patients [55,56]. However, an eventual role of the microbiota in mediating anti-tumor effects of KDs has so far not been investigated. Such a role appears possible given the results of recent studies indicating an important contribution of the gut microbiome in alleviating the symptoms of three different neurological disease conditions, namely, autism [57], multiple sclerosis [58], and infantile refractory epilepsy. In the BTBR^T+tf/j^ mouse model of autism spectrum disorder, a KD normalized the overabundance of the mucin-degrading bacterium *A. muciniphila* and significantly increased the Firmicutes/Bacteroidetes ratio which is typically low in autism spectrum disorder [57]. In patients with multiple sclerosis, a KD initially decreased total gut bacterial concentrations during the first weeks but, when maintained over six months, was able to restore the microbial biofermentative mass to levels similar to those in healthy controls [58]. Finally, in infants with refractory epilepsy, a KD decreased seizure frequency in the majority of cases and significantly changed the gut microbial composition towards that of healthy controls: *Bacteroides* and *Prevotella* increased, while *Cronobacter* levels decreased by approximately 50% [59]. Together, these three studies indicate that a KD is able to reverse the dysbiosis associated with diverse neurological disorders. If such beneficial changes can also be achieved in cancer patients remains to be tested.

## 7. Paleolithic Diet, Microbiota, and Cancer

Evolutionary medicine provides a framework for a common explanation of the increase of NCDs, according to which an insufficient adaption to the modern lifestyle leads to disease. Diet plays a key role within this framework, and the term “Paleolithic diet” or “Paleo diet”, (PD) has been introduced to refer to a modern diet mimicking the diet of our ancestors during the Old Stone Age, which chronologically spans the majority of human existence [60]. The modern PD typically consists of the following patterns: (a) High consumption of fruits, herbs, spices, and vegetables; (b) Moderate-to-high consumption of lean meats, organs, fish, and eggs; (c) Moderate consumption of nuts and seeds; (d) Exclusion of all processed foods, legumes, grains, dairy products, and plant oils (except for olive and coconut oil) and, in some PD variants, also of nightshades. It is the latter characteristic (d) that distinguishes the PD from most other diets and could be part of an explanation for the superiority of a PD over other “healthy” diets (including the MD) in small randomized trials [61,62]; in particular, grains and legumes have been causally associated with the disruption of intestinal barrier integrity and the promotion of auto-immune and inflammatory NCDs, including cancer and obesity [61,63,64]. Indeed, several protocols for treating auto-immune diseases exist, which are based on PD eating patterns, focusing on the elimination of several food groups typical of the Western diet [65,66]. A PD also ensures a high consumption of microbiota-accessible carbohydrates, which is predicted to optimize gut microbial diversity [67]. In fact, the microbiome of the Tanzanian Hadza hunter–gatherers, who still consume a diet mostly composed of foods that would have been available to early humans during the Paleolithic era in Africa, has been shown to be much more diverse than that of urban Italians, with Firmicutes (72 ± 1.9%) and Bacteroidetes (17 ± 1.1%) dominating on the phylum level, followed by Proteobacteria (6 ± 1.2%) and Spirochaetes (3 ± 0.9%), which were relatively enriched compared to Italians [68]. However, the evolutionary perspective would also entail that the Hadza microbiome is optimized for their lifestyle as equatorial hunter–gatherers, while the “optimal” ancestral microbiome of Europeans or other populations further away from the equator would be different, because non-equatorial hunter–gatherer diets are lower in carbohydrate and fiber [69]. A very recent study investigated healthy Italians following a modern PD for more than one year. The dominating phyla of the gut microbiome were Firmicutes (65.1 ± 2.1%) and Bacteroidetes (24.6 ± 2.2%), followed by Proteobacteria (4.4 ± 1.6%), Actinobacteria (3.4 ± 0.8%), and Verrucomicrobia (1.2 ± 0.5%). At the family level, the dominant bacteria were Ruminococcaceae, Lachnospiraceae, Bacteroidaceae, and Prevotellaceae, while, at the genus level, *Bacteroides*, *Pervotella*, and *Faecalibacterium* dominated [70]. Compared to Italians adhering to the MD, the PD microbiome diversity was much higher and comparable to that of the Hadza hunter–gatherers. This study is important in showing that the loss of microbiome diversity in Western societies can be counteracted by returning to a modern PD composed of natural (also region-specific) foods but without dairy, grains, refined sugar, and other processed foods. The association with high microbiome diversity and their putatively anti-inflammatory properties would make PDs interesting to investigate in future clinical studies as adjuncts to cancer therapy. Recently, a Hungarian group published several case reports of a ketogenic version of a PD appearing therapeutic against tumor growth [71,72,73], demonstrating the potential of this approach.

## 8. Conclusions

The dietary patterns discussed in this review are emerging to be potentially effective for preventing cancer and increasing the overall health of individuals. These effects are mediated, at least in part, by the promotion of an eubiotic microbiome. There is also evidence that such dietary modulation of the microbiota could have synergistic effects during cancer therapy, although it has to be acknowledged that most research in this area so far relies on mouse models whose microbiota, although similar at the phylum level, are slightly different at lower taxonomic levels. Clinical studies are therefore needed to confirm the role of microbiota modulation in cancer treatment, as suggested by these animal data. Nevertheless, the diffusion of the “fast food culture” in industrialized countries and its hallmark, the Western diet, which is low in fiber and rich in sugar and processed foods, are tightly linked to a loss of microbial diversity, dysbiosis, and a high risk of obesity, cardiovascular diseases, metabolic syndrome, and cancer. While eubiosis may heavily depend on the environment of an individual and may therefore be hard to characterize in terms of general microbial composition, there is strong evidence that individual microbiomes would benefit from efforts towards spreading and recommending a new food culture based on limiting dietary surplus and on preferring natural, regional, and fresh foods.
